# Urinary sodium indices do not reliably differentiate stage B2 myxomatous mitral valve disease in dogs from healthy dogs

**DOI:** 10.3389/fvets.2026.1777916

**Published:** 2026-05-22

**Authors:** Minsuk Kim, Yeongseo Park, Chul Park

**Affiliations:** Department of Veterinary Internal Medicine, College of Veterinary Medicine, Jeonbuk National University, Iksan, Republic of Korea

**Keywords:** echocardiography, myxomatous mitral valve disease, renin-angiotensin-aldosterone system, stage B2, urinary sodium

## Abstract

**Introduction:**

Myxomatous mitral valve disease (MMVD) is associated with neurohormonal dysregulation, but practical markers for routine clinical use remain limited. This study evaluated whether spot urine sodium indices differ between healthy dogs and dogs with stage B2 MMVD and whether they are associated with echocardiographic indices of disease severity.

**Methods:**

In this prospective, single-center, cross-sectional study, 44 healthy dogs and 18 dogs with MMVD stage B2 were enrolled. Spot urine samples collected during routine visits were analyzed for urinary sodium concentration (uNa), urinary potassium concentration, urinary sodium-to-potassium ratio (uNa/uK), and urine-specific gravity (USG)-corrected uNa. Associations between urinary sodium indices and echocardiographic parameters, including LA:Ao, LVIDdN, and E peak, were assessed.

**Results:**

Urine sodium indices showed substantial inter-individual variability and did not differ significantly between healthy dogs and dogs with MMVD stage B2. Within the stage B2 group, none of the urine sodium indices were significantly associated with LA:Ao, LVIDdN, or E peak.

**Discussion:**

Spot urine sodium indices obtained under routine clinical conditions did not reliably differentiate dogs with stage B2 MMVD from healthy dogs and may have limited utility as standalone surrogates for neurohormonal status in preclinical MMVD.

## 1 Introduction

Myxomatous mitral valve disease (MMVD) is the most common acquired heart disease in dogs (1–3). In veterinary clinical practice, the diagnosis, staging, and treatment of MMVD are generally based on the American College of Veterinary Internal Medicine (ACVIM) consensus guidelines (1). A survey conducted in the U.S. on the management of MMVD in dogs found that angiotensin converting enzyme inhibitors (ACEi) were the most common monotherapy for dogs with stage B2 before the EPIC trial. After the trial, pimobendan use increased markedly, both as monotherapy and in combination with ACEi. Nevertheless, even post-EPIC, about 9% of referring veterinarians and 60% of cardiologists continued to prescribe ACEi for MMVD stage B2 dogs (4, 5). A recent survey conducted in the Netherlands reported that the prescription rate of ACEi in dogs with stage B2 MMVD was 4% (6). Although regional differences exist, a considerable number of veterinarians still prescribe ACEi for dogs with MMVD stage B2. In clinical practice, ACEi prescribing in dogs with stage B2 MMVD is often based on clinician judgment rather than individualized assessment of renin-angiotensin-aldosterone system (RAAS) activity. Given the marked inter-individual variability in RAAS activation, further work is needed to determine whether RAAS guided ACEi use could improve outcomes in selected dogs (7).

MMVD is associated with neurohormonal dysregulation, including activation of the RAAS, which may contribute to sodium and water retention and pathologic remodeling (7–12). The degree of RAAS activation may vary among individual dogs, even within the same disease stage (13, 14). To date, most studies have evaluated treatment effects according to disease stage without considering the individual variability in RAAS activation among dogs. The most conventional marker currently used to assess RAAS activation is the urine aldosterone-to-creatinine ratio (UAldo:C). In addition, circulating renin levels, angiotensin peptides, and aldosterone concentrations can be measured to evaluate RAAS activity (7, 11, 13, 15, 16). However, these measurements typically require submission to external laboratories, and the collection and handling of samples, particularly for renin and angiotensin peptides, are technically challenging, limiting their routine use in clinical practice.

In contrast, urine sodium (uNa) and related indices can be easily measured in routine clinical settings and may serve as a functional readout of renal sodium handling (17). However, spot uNa is inherently variable and is influenced by multiple physiologic and external factors, including dietary sodium intake, hydration status, age, breed, timing of feeding and water intake, and urine collection conditions (13, 18). Although RAAS activation could theoretically reduce urinary sodium excretion under controlled conditions, uNa is not a specific marker of RAAS activity and should be interpreted cautiously. To date, uNa has primarily been investigated in dogs with stage C MMVD, largely in the context of assessing natriuretic response to diuretic therapy, whereas its performance and variability in preclinical stage B2 MMVD have not been characterized (17).

The objectives of this study were to (1) determine whether uNa-related indices—urinary sodium concentration (uNa), the urinary sodium-to-potassium ratio (uNa/uK), and corrected uNa (as defined in the Methods) differ between healthy dogs and those with stage B2 MMVD and to characterize their within-stage variability in spot urine samples obtained under routine clinical conditions and (2) evaluate the associations between these uNa-related indices and echocardiographic indices of MMVD severity, including the left atrium-to-aorta ratio (LA:Ao), diastolic left ventricular normalized dimensions (LVIDdN), and peak velocity of early diastolic transmitral flow (E peak).

## 2 Materials and methods

### 2.1 Animals

This study was a prospective, observational cross-sectional study approved by the Institutional Animal Care and Use Committee of Jeonbuk National University (Approval No. NON2025-186-001). The study was conducted on client-owned dogs presented to the Jeonbuk Animal Medical Center between November 2024 and September 2025. Owner consent was obtained for all dogs prior to enrollment.

All dogs underwent a screening evaluation before enrollment, including history taking, physical examination, blood pressure measurement, and serum biochemistry analysis including the SNAP 4Dx Plus test. Dogs with MMVD were assigned to the MMVD stage B2 group if they met the ACVIM consensus criteria: murmur intensity ≥3/6, LA:Ao ≥ 1.6, LVIDdN ≥1.7, and vertebral heart score (VHS) ≥ 10.5. Pimobendan use was allowed in the MMVD stage B2 group, but only at doses ≤0.25 mg/kg PO every 12 h; dogs receiving higher doses were excluded.

Dogs with systemic illness or with conditions such as heartworm disease, congenital cardiac disease, or pulmonary hypertension, as well as other cardiovascular disorders, were excluded. Additional exclusion criteria included a history of polyuria/polydipsia, systolic blood pressure ≥160 mmHg, and serum creatinine concentration ≥2.0 mg/dL. Dogs that had received ACEi, angiotensin receptor blockers, diuretics, spironolactone, corticosteroids, sympathomimetics and beta-blockers within the preceding 3 months were also excluded.

### 2.2 Sample collection and analysis

To reflect routine clinical practice, urine samples were obtained as spot samples during routine clinic visits. Samples were obtained via free catch or cystocentesis. For cystocentesis, dogs were gently restrained in dorsal or lateral recumbency during sample acquisition. Urine-specific gravity (USG), urinary sodium and potassium concentrations were measured immediately after sample collection. Urinalysis including sediment examination was performed, and dogs with evidence of urinary tract infection were excluded. USG was measured using a Reichert VET360 refractometer, and urinary electrolyte concentrations were measured using a FUJIFILM DRI-CHEM NX700V analyzer. The calculated urinary sodium indices were as follows:

- Urinary sodium concentration (mmol/L).
- USG-corrected urinary sodium concentration (mmol/L).

o uNa_measured × [(USG_normal−1.000)/(USG_measured−1.000)].
o USG_normal = 1.030.

- Urinary sodium-to-potassium ratio.

To account for urine dilution in spot samples, we used USG, a readily measured clinical parameter, to normalize uNa for urine concentration, thereby generating the USG-corrected urinary sodium concentration, by referencing a USG of 1.030 as a representative value for adequately concentrated canine urine (19).

### 2.3 Echocardiography

Echocardiography was performed using a Philips EPIQ 7C ultrasound unit by an experienced veterinarian (M.K.). Standard 2-dimensional, M-mode, and Doppler images were obtained from right and left parasternal windows. Echocardiographic measurements included the LA:Ao, LVIDdN, and E peak. Each index was measured three times, and the mean value was used for analysis (20–22). The echocardiographer was blinded to urinary electrolyte results at the time of measurement. Representative echocardiographic images illustrating the measurement methods for LA:Ao, LVIDd, and E peak are provided in Supplementary Figure S1.

### 2.4 Statistical analysis

All statistical analyses were performed using IBM SPSS Statistics, version 29.0 (IBM Corp., Armonk, NY, United States). The Shapiro–Wilk test was used to assess normality. Variables with a normal distribution were reported as means ± standard deviations, whereas non-normally distributed variables were presented as medians and interquartile range (IQR).

Comparisons of signalment variables between groups were assessed using independent samples *t*-tests, Mann–Whitney U tests, and Pearson’s chi-square tests, as appropriate. Comparisons of urinary sodium indices between groups were evaluated using the Mann–Whitney U test. Correlations between urinary sodium indices and echocardiographic variables, as well as correlations among the urinary sodium indices themselves, were assessed using Spearman correlation coefficients. Statistical significance was set at *p* < 0.05.

## 3 Results

A total of 62 dogs were included in the study, comprising 44 control dogs and 18 dogs in the MMVD stage B2 group. Breeds represented in the study included mixed-breed dog (*n* = 13), Poodle (*n* = 15), Cocker Spaniel (*n* = 4), Beagle (*n* = 3), Shih Tzu (*n* = 3), Maltese (*n* = 2), Jindo (*n* = 2), Dachshund (*n* = 2), Bichon Frise (*n* = 1), Spitz (*n* = 1), Yorkshire Terrier (*n* = 1), Chihuahua (*n* = 1), Pekingese (*n* = 1), Brittany (*n* = 1), and Siberian Husky (*n* = 1). The mean age was 8.4 ± 4.8 years in the control group and 12.1 ± 2.6 years in the MMVD stage B2 group. Median body weight was 6.8 kg (5.1–10.4 kg) in controls and 5.2 kg (4.4–7.5 kg) in the MMVD stage B2 group. The control group consisted of 18 males and 26 females, whereas the MMVD stage B2 group consisted of 9 males and 9 females; sex distribution did not differ significantly between groups (*p* = 0.512). Dogs with MMVD stage B2 were significantly older than control dogs (*p* < 0.001). Body weight tended to be lower in the MMVD stage B2 group compared with controls, showing a borderline statistically significant difference (*p* = 0.050). Among the 18 dogs with MMVD stage B2, 15 were receiving pimobendan at the time of enrollment. The MMVD stage B2 group showed a mean VHS of 11.5 ± 0.8, a median LA:Ao of 1.9 (1.7–2.2), a mean LVIDdN of 1.9 ± 0.2, and a mean E peak of 1.2 ± 0.2 m/s. Signalment, radiographic, and echocardiographic characteristics of the control and MMVD stage B2 groups are summarized in Table 1.

**Table 1:** Signalment, radiographic, and echocardiographic parameters of control and MMVD stage B2 groups.

Variable	Control (*n* = 44)	MMVD stage B2 (*n* = 18)	*p*-value
Age (years)	8.4 ± 4.8	12.1 ± 2.6	<0.001
Body weight (kg)	6.8 (5.1–10.4)	5.2 (4.4–7.5)	0.050
Sex (M/F; %)	(18/26) (59.1/40.9)	(9/9) (50, 50)	0.512
VHS	–	11.5 ± 0.8	–
LA:Ao	–	1.9 (1.7–2.2)	–
LVIDdN	–	1.9 ± 0.2	–
E peak (m/s)	–	1.2 ± 0.2	–

VHS, vertebral heart size; LA:Ao, left atrial-to-aortic ratio; LVIDdN, left ventricular internal diameter in diastole, normalized for body weight; E peak, peak velocity of early diastolic transmitral flow.

**Figure 1:**
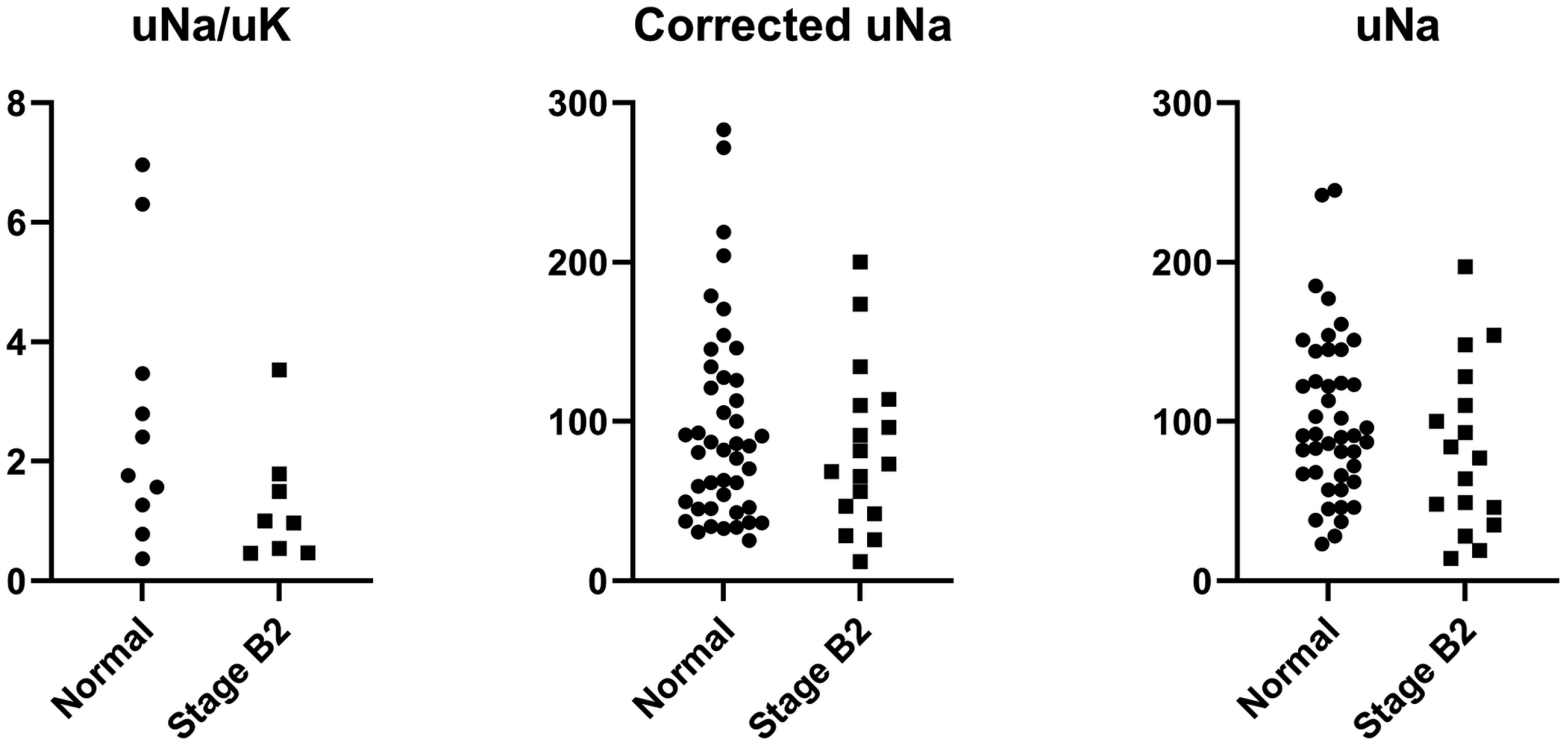
Dot plots of urinary sodium indices, including uNa, USG-corrected uNa, and uNa/uK, comparing normal dogs and dogs with MMVD stage B2. The units for uNa and USG-corrected uNa are mmol/L.

Comparisons of urinary sodium indices between the control and MMVD stage B2 groups are summarized in Table 2. One dog in the MMVD stage B2 group did not have a measurable uNa and was excluded from analyses involving this variable. Urinary potassium concentration (uK) was available for 10 control dogs and 8 dogs in the MMVD stage B2 group; therefore, only these dogs were included in analyses of the uNa/uK. uNa did not differ significantly between control dogs (median, 91.0 mmol/L; 66.3–139.3 mmol/L) and dogs with MMVD stage B2 (median, 77.0 mmol/L; 40.5–119.0 mmol/L; *p* = 0.182). Similarly, USG-corrected uNa showed no significant difference between the control group (median, 83.4 mmol/L; 45.5–127.1 mmol/L) and the MMVD stage B2 group (median, 73.4 mmol/L; 44.4–111.9 mmol/L; *p* = 0.584). The uNa/uK also did not differ significantly between controls (median, 2.1; 1.1–4.3) and dogs with MMVD stage B2 (median, 1.0; IQR, 0.5–1.7; *p* = 0.146). These distributions are illustrated in Figure 1. Overall, none of three urinary sodium indices evaluated in this study differed significantly between the MMVD stage B2 and control groups.

**Table 2:** Comparison of urinary sodium indices between control and MMVD stage B2 groups.

Variable	Control		MMVD stage B2		*p*-value
	*N*	Median (IQR)	*N*	Median (IQR)	
uNa (mmol/L)	44	91.0 (66.3–139.3)	17	77.0 (40.5–119.0)	0.182
USG-corrected uNa (mmol/L)	44	83.4 (45.5–127.1)	17	73.4 (44.4–111.9)	0.584
uNa/uK	10	2.1 (1.1–4.3)	8	1.0 (0.5–1.7)	0.146

uNa, urinary sodium concentration; uNa/uK, Urinary sodium/potassium ratio.

Results of the correlation analyses between urinary sodium indices and echocardiographic parameters are presented in Table 3. Correlations were performed using the available sample size for each variable. uNa was not significantly correlated with either the LA:Ao (r = −0.163, *p* = 0.533), LVIDdN (r = 0.052, *p* = 0.844), or E peak (r = 0.280, *p* = 0.277). Similarly, the USG-corrected uNa showed no significant correlation with the LA:Ao (r = 0.124, *p* = 0.635), LVIDdN (r = 0.101, *p* = 0.701), or E peak (r = 0.143, *p* = 0.583). The uNa/uK also demonstrated no significant correlation with the LA:Ao (r = 0.364, *p* = 0.376), LVIDdN (r = 0.095, *p* = 0.823), or E peak (r = 0.214, *p* = 0.610) Overall, none of the three urinary sodium indices evaluated in this study showed a significant association with any of the echocardiographic parameters in dogs with MMVD stage B2.

**Table 3:** Spearman correlation coefficients between urinary sodium indices and echocardiographic parameters in dogs with MMVD stage B2.

Variable	Spearman correlation coefficient; *p*-value		
	LA:Ao	LVIDdN	E peak (m/s)
uNa (mmol/L)	−0.163; 0.533	0.052; 0.844	0.280; 0.277
USG-corrected uNa (mmol/L)	0.124; 0.635	0.101; 0.701	0.143; 0.583
uNa/uK	0.364; 0.376	0.095; 0.823	0.214; 0.610

Sample sizes: uNa (*n* = 17), USG-corrected uNa (*n* = 17), uNa/uK (*n* = 8). uNa, urinary sodium concentration; uNa/uK, Urinary sodium/potassium ratio; LA:Ao, left atrial-to-aortic ratio; LVIDdN, left ventricular internal diameter in diastole, normalized for body weight; E peak, peak velocity of early diastolic transmitral flow.

Correlation analyses among the urinary sodium indices were performed using all available samples from the control and MMVD stage B2 groups. The analyses were conducted based on the available sample size for each variable, and the results are summarized in Table 4. uNa and USG-corrected uNa were very strongly positively correlated (r = 0.829, *p* < 0.001). uNa was also very strongly positively correlated with the uNa/uK (r = 0.884, *p* < 0.001). In addition, USG-corrected uNa showed a very strong positive correlation with the uNa/uK (r = 0.909, *p* < 0.001). Overall, the three urinary sodium indices demonstrated very high degrees of intercorrelation.

**Table 4:** Spearman correlation coefficients among urinary sodium indices in all dogs.

Variable	Spearman correlation coefficient		
	uNa (mmol/L)	USG-corrected uNa (mmol/L)	uNa/uK
uNa (mmol/L)	–	0.829	0.884
USG-corrected uNa (mmol/L)	0.829	–	0.909
uNa/uK	0.884	0.909	–

All correlation coefficients were statistically significant (*p* < 0.001). Sample sizes: uNa (*n* = 61), USG-corrected uNa (*n* = 61), uNa/uK (*n* = 18). uNa, urinary sodium concentration; uNa/uK, Urinary sodium/potassium ratio.

## 4 Discussion

This study investigated differences in uNa, uNa/uK, and corrected uNa between healthy dogs and those with MMVD stage B2, and further examined the relationships between urine sodium indices and echocardiographic indices of cardiac remodeling within the stage B2 population. As a result, none of the three indices showed statistically significant differences between healthy dogs and those with stage B2 disease. Moreover, within the stage B2 dogs, no meaningful correlations were identified between urine sodium indices and echocardiographic indices, including LA:Ao, LVIDdN and E peak. Additionally, although direct comparison with a gold standard was not available, the wide inter-individual variability observed in all three indices, as shown by their median and IQR ranges, suggests substantial fluctuation even within the same MMVD stage. This finding implies that the degree of RAAS activation may be influenced by multiple factors at the time of measurement, including diet, hydration status, glomerular filtration rate, and other physiological variables (13, 14, 18). Therefore, under routine outpatient conditions, spot urinary sodium indices appear insufficient for individual-level decision-making regarding neurohormonal status or RAAS-guided therapy in MMVD stage B2.

In this study, uNa, uNa/uK, and corrected uNa showed no significant differences between the two groups. This finding suggests two possible interpretations. First, it is possible that RAAS activation may not be consistently upregulated in dogs with MMVD stage B2. Previous evidence has indicated that in dogs with MMVD stage B2, activation of the classical RAAS pathway may not occur, whereas the alternative RAAS pathway, characterized by increased angiotensin converting enzyme 2 (ACE2) activity, may be preferentially upregulated (7). Theoretically, uNa and related urine sodium indices decrease in the presence of classical RAAS activation, whereas activation of the alternative RAAS pathway, particularly enhanced ACE2 activity, would be expected to increase these values. A second possibility is that there is considerable inter-individual variability in RAAS activation. Prior studies have shown that plasma renin and aldosterone concentrations in dogs with stage B2 span a wide range, indicating that the degree of RAAS activation may differ substantially among individuals even within the same disease stage (7). Similarly, previous investigations in dogs with CHF have demonstrated broad dispersion in uNa measurements, further supporting that urine sodium indices may reflect heterogeneous physiologic states across patients (10). In the present study, uNa values also exhibited a wide distribution across individuals, likely influenced by uncontrolled factors. Although not statistically significant, Figure 1 shows that dogs with stage B2 tended to have lower uNa-related indices. This pattern, together with the marked inter-individual variability, implies that only a subset of B2 dogs may exhibit meaningful RAAS activation. Identifying these individuals and comparing their uNa indices with other established markers of RAAS activity would be valuable in future studies.

When the correlations between uNa-related indices and echocardiographic variables were evaluated, no significant correlations were observed. As noted above, the wide range and substantial variability of these indices, along with inter-individual differences in the degree of RAAS activation, may have obscured potential associations with MMVD severity (10, 13, 23). Additionally, the stage B2 population is heterogeneous, and it remains possible that significant associations might emerge if analyses were restricted to dogs with more advanced B2 disease, such as those classified using the MINE score 2 (24). In addition, RAAS activation represents only one component of the compensatory mechanisms involved in the progression of MMVD. Other systems, including the sympathetic nervous system and natriuretic peptides, also contribute to hemodynamic adaptation. Integrating urine sodium indices with these neurohormonal pathways, alongside echocardiographic markers of MMVD severity, may provide a more comprehensive understanding of the underlying pathophysiology (25).

The correlation analysis among the urine indices revealed a strong association between corrected uNa and uNa/uK. Because uNa is highly susceptible to dilutional effects and may therefore be limited in its ability to reflect physiologic states accurately, uNa/uK is commonly used as a corrective measure to account for this variability (26). In this study, we generated a corrected uNa value by adapting a method used in human medicine that incorporates urine specific gravity into sodium normalization (27). Correlation analysis demonstrated a very strong association between corrected uNa and uNa/uK. These findings suggest that corrected uNa may serve as a practical alternative to uNa/uK in situations where uK cannot be measured reliably or when sample volume is insufficient. Further studies are warranted to validate this potential application.

This study has several limitations. First, the sample size was relatively small. Because the stage B2 population is heterogeneous, further stratification using metrics such as the MINE score 2 may allow more precise evaluation of RAAS activation within this group. Second, physiologic factors that can influence urine sodium indices, including dietary sodium intake, feeding time, water consumption, and the timing of urine collection, were not controlled. Third, we did not concurrently measure gold-standard RAAS markers such as plasma renin and aldosterone concentrations or angiotensin peptide profiles, which limited our ability to directly validate how accurately the urine-derived indices reflect true RAAS activity. Additionally, a portion of uK measurements failed because potassium quantification was performed in an off-label manner using the NX700, resulting in a reduced sample size for uK. Finally, because this study employed a cross-sectional design, temporal changes in RAAS activation or their relationship to disease progression within stage B2 could not be assessed.

In conclusion, spot urinary sodium indices (uNa, uNa/uK, and USG-corrected uNa) obtained under routine clinical conditions did not reliably differentiate dogs with stage B2 MMVD from healthy dogs and were not associated with echocardiographic markers of remodeling. These findings suggest that single time-point urine sodium metrics, when collected without standardization of diet and sampling conditions, should not be used as surrogates of neurohormonal activation in preclinical MMVD. Future studies incorporating standardized intake, repeated sampling, and concurrent RAAS biomarkers are warranted to clarify whether specific neurohormonal phenotypes can be identified within stage B2 disease.

## Data Availability

The raw data supporting the conclusions of this article will be made available by the authors, without undue reservation.

## Supplementary material

SUPPLEMENTARY FIGURE S1 Representative echocardiographic images illustrating key measurements used in this study. **(A)** Measurement of the left atrial-to-aortic root ratio (LA:Ao) from the right parasternal short-axis view. **(B)** M-mode measurement of the left ventricular internal diameter in diastole (LVIDd), which was used to calculate normalized LVIDd (LVIDdN). **(C)** Pulsed-wave Doppler tracing of transmitral inflow showing measurement of peak early diastolic velocity (E peak). These images are provided to demonstrate the echocardiographic measurement techniques applied in the study.
