# Supplementary figures and images for: Urinary sodium indices do not reliably differentiate stage B2 myxomatous mitral valve disease in dogs from healthy dogs
Source: Front Vet Sci. 2026 May 22;13:1777916. doi: 10.3389/fvets.2026.1777916 (PMC13236519; doi:10.3389/fvets.2026.1777916)

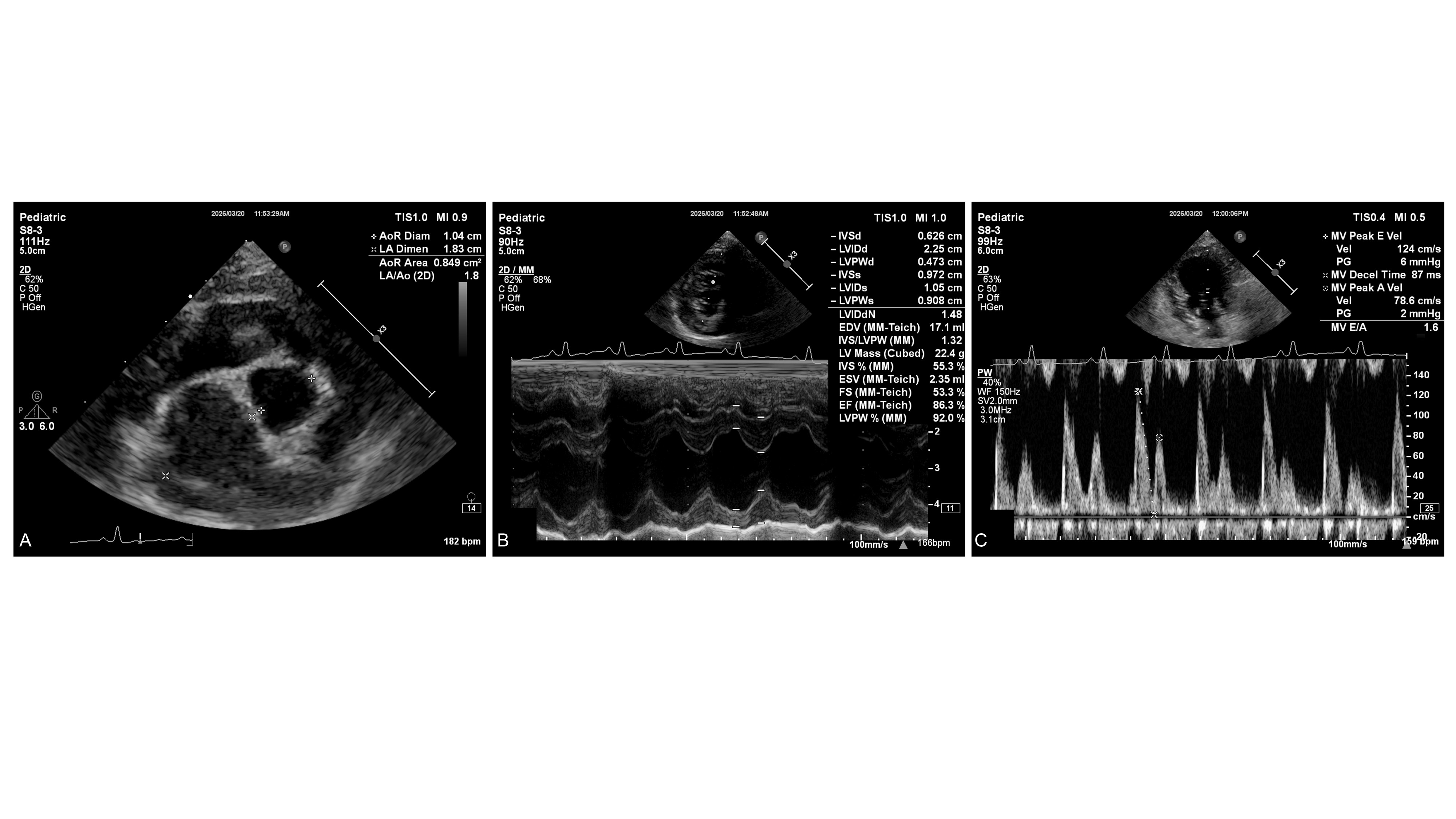

Supplement: SUPPLEMENTARY FIGURE S1 — Representative echocardiographic images illustrating key measurements used in this study. (A) Measurement of the left atrial-to-aortic root ratio (LA:Ao) from the right parasternal short-axis view. (B) M-mode measurement of the left ventricular internal diameter in diastole (LVIDd), which was used to calculate normalized LVIDd (LVIDdN). (C) Pulsed-wave Doppler tracing of transmitral inflow showing measurement of peak early diastolic velocity (E peak). These images are provided to demonstrate the echocardiographic measurement techniques applied in the study. [file Image_1.TIF]
